# Supplementary material for: Content-rich biological network constructed by mining PubMed abstracts
Source: BMC Bioinformatics. 2004 Oct 8;5:147. doi: 10.1186/1471-2105-5-147 (PMC528731; doi:10.1186/1471-2105-5-147)
Supplement: Additional File 5 — The original Chilibot query results of the term "long-term potentiation (LTP)" and 22 other terms, limiting the latest references analyzed to the years 1990, 1995, 2000, and 2004. [file 1471-2105-5-147-S5.bz2 › chilibotAdditionalFile5/ltp1995/html/PKA_ACTIN.html]

 


 **PKA** and **ACTIN** 
  
Found 16 abstracts in PubMed,  **16 abstracts were retrieved and analyzed**.  


---

 Search Google  |
 PDF files only 
|  EDU domain only 

---

**Interactive relationship** (e.g. stimulation, inhibition, etc)

- Protein kinase A  **PKA**  activation of epithelial Na channels requires  **actin**  filaments.  Ref: 7541942 Am J Physiol, 1995
- The results demonstrate that phosphorylation of dystrophin by  **PKA**  phosphorylation caused a three fold increase in dystrophin binding to  **actin** .  Ref: 7818551 Biochem Biophys Res Commun, 1995
- Disruption of endogenous  **actin**  filament organization with cytochalasin D for at least 1 h prevented the  **PKA**  mediated activation of Na channels but not activation following the addition of  **actin**  to the cytosolic side of the patch.  Ref: 8393280 Am J Physiol, 1993
- When compared with wild type metastatic LLC LN7 cells, in which the F  **actin**  staining was too diffuse to be clearly visualized microscopically, the  **PKA**  defective REV LN7 transfectants had an increased level of F  **actin** .  Ref: 8314333 Int J Cancer, 1994
- These data suggest that the form of  **actin**  required for Na channel activationi.e., short  **actin**  filaments may be favored by the phosphorylation of G  **actin**  and may thus mediate or facilitate the activation of Na channels by  **PKA** .  Ref: 8393280 Am J Physiol, 1993
- We conclude that beta adrenergic stimulation increases the rate of crossbridge release from  **actin** , by a mechanism that most likely involves the phosphorylation of troponin I and or C protein by  **PKA** .  Ref: 8118962 Circ Res, 1994
- **Actin**  was phosphorylated by  **PKA**  with an equilibrium stoichiometry of 2 1 mol PO4  **actin**  monomer.  Ref: 8393280 Am J Physiol, 1993
- To assess the role of  **PKA**  on  **actin**  filament organization,  **actin**  was used as a substrate for the specific phosphorylation by the  **PKA** .  Ref: 8393280 Am J Physiol, 1993
- In excised inside out patches, addition of deoxyribonuclease I, which prevents  **actin**  polymerization, inhibited Na channel activation mediated by  **PKA** .  Ref: 8393280 Am J Physiol, 1993
- In comparison with the wild type non metastatic LLC C8 cells, which had a high content of F  **actin** , the CEV C8 transfectants that over expressed  **PKA**  activity had a reduced level of F  **actin** .  Ref: 8314333 Int J Cancer, 1994

**Parallel relationship** (e.g. studied together, co-existance, homology, etc.)

- When Ddpk3 null cells are complemented with DdPK3 expressed from an  **actin**  promoter on an extrachromosomal vector low copy number,  **PKA**  activity is restored and the cells proceed to the slug stage but will not culminate, suggesting that properly regulated  **PKA**  activity is essential for culmination.  Ref: 1332055 Proc Natl Acad Sci U S A, 1992
- In contrast, non metastatic LLC C8 tumor cells, which have a diminished level of  **PKA**  signaling, do not produce GM CSF and do not respond to exogenous GM CSF, since they remain non motile and retain a high content of filamentous  **actin** .  Ref: 8314333 Int J Cancer, 1994
- Thus  **actin**  allowed to polymerize for at least 1 h in the presence of  **PKA**  and ATP to obtain phosphorylated  **actin**  filaments induced Na channel activity in excised inside out patches, in contrast to  **actin**  polymerized either in the absence of  **PKA**  or in the presence of  **PKA**  plus a  **PKA**  inhibitor nonphosphorylated  **actin**  filaments.  Ref: 8393280 Am J Physiol, 1993
- In the present study, the role of  **actin**  in the activation of apical Na channels by the adenosine cyclic monophosphate dependent protein kinase A  **PKA**  was investigated by patch clamp techniques in A6 epithelial cells.  Ref: 8393280 Am J Physiol, 1993
- We investigated the cause of the aggregation defect in transformants overexpressing dominant negative  **PKA**  regulatory subunits  **PKA**  RM under an  **actin**  15 promoter.  Ref: 7628643 FEBS Lett, 1995
- The data indicate that CFTR is regulated by  **actin**  filaments whose effect may, in turn, be associated with the  **PKA**  dependent pathway.  Ref: 7541942 Am J Physiol, 1995
